# Expanding ISME Communications to taxonomy and nomenclature

**DOI:** 10.1093/ismeco/ycaf106

**Published:** 2025-06-30

**Authors:** Maria Chuvochina, Marike Palmer, Stephanus N Venter

**Affiliations:** Leibniz Institute DSMZ-German Collection of Microorganisms and Cell Cultures, Inhoffenstraße 7 B, Braunschweig 38124, Germany; Department of Microbiology, University of Manitoba, 66 Chancellor's Circle, Winnipeg, MB, R3T 2N, Canada; Department of Biochemistry, Genetics and Microbiology, and Forestry and Agricultural Biotechnology Institute (FABI), University of Pretoria, Lynnwood Road, Pretoria, 0002, South Africa

Research in microbial ecology is often accompanied by the classification and naming of new taxa, typically included as part of the results section of an ecological study. Bold and beautiful, Latin names are more than labels—they are essential tools for scientific communication. Nested within a hierarchical system, they populate public databases and enable researchers to search for organisms, retrieve associated sequences and metadata, and explore related taxa across various taxonomic levels. In recognition of the vital role that naming and classification play in ecological studies, ISME Communications is proud to introduce Taxonomy and Nomenclature as part of its scope.

## Why do we need to include this topic in the journal?

One might argue that there are already enough journals dedicated to taxonomic research and the descriptions of new taxa such as the *International Journal of Systematic and Evolutionary Microbiology, Systematic and Applied Microbiology*, or *FEMS Microbiology Letters*. However, most papers submitted to these journals typically deal with cultured taxa and focus on taxonomic descriptions rather than ecology. Recent advances in sequencing technologies, metagenomics, and transitions toward genome-based taxonomies, have led to an explosion in the discovery of novel microbial diversity. The majority of this diversity is as-yet-uncultured and is either unnamed or known only through alphanumeric placeholder names. This represents a massive naming challenge, as naming plays a crucial role in cataloguing and communicating information about microbial diversity. According to the Genome Taxonomy Database release 09-RS220, around 90 000 prokaryotic species remain unnamed. For many years, authors have been able to provide names only through a provisional route, *Candidatus* names, which lack formal standing in nomenclature and regulations. This practice eventually led to chaos because effective scientific communication and consistent application of such names in taxonomic frameworks was highly problematic. One key shortfall in how the community used *Candidatus* names was the practice of naming higher taxonomic ranks without designating and naming nomenclatural types for those taxa. In the absence of nomenclatural types to anchor *Candidatus* names, classification can be compromised, e.g. if the group becomes polyphyletic in gene or genome trees. In these cases, ambiguity in terms of which organisms should be associated with which names become highly complex, especially during reclassification efforts. Consequently, there was a growing sense that an urgent solution was needed to address these problems.

A solution has come after long debates among traditional and “neo”-taxonomists, following the official rejection of genome sequences as types by the International Committee on Systematics of Prokaryotes (ICSP). That solution is called the Code of Nomenclature of Prokaryotes Described from Sequence Data, or SeqCode for short. The ISME Society, recognizing the need of its membership to provide valid Latin names to uncultured taxa, has supported the SeqCode initiative, a nomenclatural code for prokaryotes described from sequence data, and its dedicated online registry for deposition and validation of names—the SeqCode Registry (https://registry.seqco.de/). At the time of writing, more than 850 names have been validated under the SeqCode. The code provides clear guidelines for naming uncultured taxa based on genome sequences, including minimum standards for genome quality and criteria for valid publications. Moreover, SeqCode recognizes names that are validly published under the International Code on Nomenclature of Prokaryotes (ICNP), thus, reducing the risk of introducing multiple synonyms. Fortunately, the creation of SeqCode motivated the ICSP to acknowledge the nomenclatural issues associated with naming of uncultured taxa which recently resulted in approval of Section 10 of the ICNP. This new section in the ICNP aims to regulate naming of uncultured taxa by establishing “pro-status” for *Candidatus* names, albeit not yet giving them full recognition. It is hoped that eventually the two Codes will unite to provide unambigous nomenclature for all prokaryotes regardless of their cultivation status.

We believe that it is our mission to promote excellence in microbial ecology research, and efficient communication of microbial diversity is essential for this. This mission is especially important in the age of big sequence data, where organization of biodiversity knowledge recovered from sequencing and binning efforts is now a routine practice in many laboratories. At the same time, we acknowledge that addressing taxonomic and nomenclatural issues can be complex and may fall outside the expertise of many microbial ecologists. Furthermore, we invite authors dealing with all domains of life but since most of the microbial diversity is prokaryotic, our focus here is primarily on the prokaryotic domains. As the Senior Editors, we aim to support the community by promoting best practices and fostering knowledge exchange, facilitating future research in microbial ecology.

The expansion of the scope to include Taxonomy and Nomenclature will cover studies in microbial ecology that include the following topics:

- Genome-based taxonomic descriptions
- Comparative taxonomic frameworks
- Methodological advances in classification
- Nomenclatural proposals

We encourage authors to register and validate names of newly proposed taxa in the SeqCode Registry or, where necessary, following the rules of ICNP. Authors may also propose names under two codes in the same paper, and we strive that our new guidelines will make this process as straightforward as possible. If pure strains are available for newly proposed taxa, we urge authors to deposit them in public culture collections to support future preservations and comparative studies. At our recent roundtable at ISME19, we saw strong interests from early career researchers in the future of prokaryotic nomenclature. We invite everyone interested in contributing to this important topic to join the SeqCode community and support this initiative. We believe that integrating and expanding our knowledge of both cultured and uncultured microbial diversity in all ecosystems, supported by proper rules of scientific communication, will ensure a stronger foundation for future studies and the unification of taxonomy and nomenclature for all prokaryotes.

